# Anatomically resectable *versus* biologically borderline resectable pancreatic cancer definition: refining the border beyond anatomical criteria and biological aggressiveness

**DOI:** 10.1093/bjsopen/zraf033

**Published:** 2025-05-20

**Authors:** Giulio Belfiori, Federico De Stefano, Domenico Tamburrino, Giulia Gasparini, Francesca Aleotti, Paolo R Camisa, Claudia Arcangeli, Marco Schiavo Lena, Nicolo Pecorelli, Diego Palumbo, Stefano Partelli, Francesco De Cobelli, Michele Reni, Stefano Crippa, Massimo Falconi

**Affiliations:** Division of Pancreatic Surgery, Pancreas Translational & Clinical Research Centre, IRCCS San Raffaele Scientific Institute, Milan, Italy; Division of Pancreatic Surgery, Pancreas Translational & Clinical Research Centre, IRCCS San Raffaele Scientific Institute, Milan, Italy; Vita-Salute San Raffaele University, Milan, Italy; Division of Pancreatic Surgery, Pancreas Translational & Clinical Research Centre, IRCCS San Raffaele Scientific Institute, Milan, Italy; Division of Pancreatic Surgery, Pancreas Translational & Clinical Research Centre, IRCCS San Raffaele Scientific Institute, Milan, Italy; Vita-Salute San Raffaele University, Milan, Italy; Division of Pancreatic Surgery, Pancreas Translational & Clinical Research Centre, IRCCS San Raffaele Scientific Institute, Milan, Italy; Division of Pancreatic Surgery, Pancreas Translational & Clinical Research Centre, IRCCS San Raffaele Scientific Institute, Milan, Italy; Vita-Salute San Raffaele University, Milan, Italy; Division of Pancreatic Surgery, Pancreas Translational & Clinical Research Centre, IRCCS San Raffaele Scientific Institute, Milan, Italy; Vita-Salute San Raffaele University, Milan, Italy; Department of Pathology, IRCCS San Raffaele Scientific Institute, Milan, Italy; Division of Pancreatic Surgery, Pancreas Translational & Clinical Research Centre, IRCCS San Raffaele Scientific Institute, Milan, Italy; Vita-Salute San Raffaele University, Milan, Italy; Department of Radiology, IRCCS San Raffaele Scientific Institute, Milan, Italy; Division of Pancreatic Surgery, Pancreas Translational & Clinical Research Centre, IRCCS San Raffaele Scientific Institute, Milan, Italy; Vita-Salute San Raffaele University, Milan, Italy; Vita-Salute San Raffaele University, Milan, Italy; Department of Radiology, IRCCS San Raffaele Scientific Institute, Milan, Italy; Vita-Salute San Raffaele University, Milan, Italy; Department of Oncology, IRCCS San Raffaele Scientific Institute, Milan, Italy; Division of Pancreatic Surgery, Pancreas Translational & Clinical Research Centre, IRCCS San Raffaele Scientific Institute, Milan, Italy; Vita-Salute San Raffaele University, Milan, Italy; Division of Pancreatic Surgery, Pancreas Translational & Clinical Research Centre, IRCCS San Raffaele Scientific Institute, Milan, Italy; Vita-Salute San Raffaele University, Milan, Italy

## Abstract

**Background:**

The anatomically resectable pancreatic ductal adenocarcinoma treatment sequence is still debated. Heterogeneity in patient characteristics within this group may explain literature discrepancies. To overcome these limits, a biologically borderline resectable pancreatic ductal adenocarcinoma category has been analysed according to institutional criteria. The aim of this study was to examine the characteristics and outcomes of patients with biologically borderline resectable pancreatic ductal adenocarcinoma and determine whether they represent a distinct clinical and prognostic subgroup.

**Methods:**

Data from all consecutive patients who underwent surgical resection for pancreatic ductal adenocarcinoma between 2015 and 2022 were retrospectively analysed. Biologically borderline resectable disease was classified by the presence of one or more of the following: carbohydrate antigen 19-9 ≥200 U/ml, cancer-related symptoms lasting >40 days, and radiological suspicion of regional lymph node metastases at diagnosis.

**Results:**

In total, 886 patients were included in the study and divided into anatomically borderline resectable (266 patients (30%)) and anatomically resectable (620 patients (70%)), which was further divided into resectable (R; 397 patients (64%)) and biologically borderline resectable (223 patients (36%)). Neoadjuvant treatment was administered in 245 patients (92.1%) in the anatomically borderline resectable group, 82 patients (20.7%) in the R group, and 135 patients (60.5%) in the biologically borderline resectable group. After a median follow-up of 45 (95% c.i. 42 to 48) months, the median disease-specific survival in the biologically borderline resectable group was 40 months compared with 59 months in the R group (*P* < 0.001) and 40 months in the anatomically borderline resectable group (*P* = 0.570). In the upfront surgery cohort, the median disease-specific survival was worse for biologically borderline resectable patients compared with R patients (27 *versus* 54 months respectively, *P* < 0.001). Biologically borderline resectable was also independently associated with worse disease-specific survival, together with age, tumour size at diagnosis, and anatomically borderline resectable. The same, except for age, were also predictors of worse event-free survival.

**Conclusion:**

Despite their identical anatomical appearance, resectable and biologically borderline resectable pancreatic ductal adenocarcinoma represent two distinct prognostic entities, warranting separate evaluation and, potentially, different treatment approaches.

## Teaser

Patients with biologically borderline resectable pancreatic cancer—an anatomically resectable disease with carbohydrate antigen 19-9 ≥200 U/ml, lymphadenopathy, and long-lasting symptoms—have poorer overall survival compared with those with resectable disease and should be considered as a specific disease group.

## Introduction

Pancreatic ductal adenocarcinoma (PDAC) is an aggressive disease, with a 5-year survival rate of 13%, and it is expected to become the second-leading cause of cancer-related mortality by 2030^1,2^. In the setting of localized and resectable (R) disease amenable to immediate surgery, 20–30% of patients will be alive after 5 years^3–10^, with only around 11% being disease-free^11,12^. In recent years, neoadjuvant chemotherapy has allowed an increased resection rate in patients with anatomically borderline and locally advanced disease^13,14^. These promising results have prompted clinicians to propose the use of neoadjuvant treatment (NAT) in anatomically resectable (AR) tumours as well, with conflicting results^15–20^. In particular, whereas the PREOPANC trial concluded that a neoadjuvant gemcitabine-based approach is superior to upfront surgery in R and borderline resectable PDAC^15^, the recent norPACT1^21^ randomized trial did not show a survival benefit from neoadjuvant FOLFIRINOX compared with upfront surgery in the R group. These differences could be related to the heterogeneity of management strategies and treatments, as well as the significant prognostic variability within the AR category. Indeed, this group is defined by anatomical criteria only, without considering features associated with biological aggressiveness that might result in early relapse after resection. Patients exhibiting these characteristics have been classified as biologically borderline resectable (BBR)^22–24^ and their management is debated, with both upfront surgery and NAT considered as possible strategies. Katz *et al*.^23^ first introduced this clinical entity, which was subsequently recognized by a consensus conference in 2017^24^, where a definition of BBR PDAC was presented based on carbohydrate antigen 19-9 (CA19-9) levels >500 U/ml, the presence of regional lymph node metastases, and/or clinical findings suggestive of, but not definitive for, distant metastases. This consensus also considered host-related factors, such as performance status and systemic inflammatory response, introducing the concept of conditional borderline disease. However, the literature also suggests different criteria and cut-offs of CA19-9, including 1000^24,25^ and 200 U/ml^26–29^. Overall, there is significant institutional variability in the definition and management of BBR PDAC. The BBR criteria used in this study include the presence of AR PDAC with CA19-9 levels ≥200 U/ml^26–28^, symptoms likely related to the disease lasting >40 days^28^, and/or radiological signs of or cytologically proven lymph node metastases^24^. The aim of this study was to analyse the characteristics and outcomes of patients with BBR PDAC, to investigate whether they represent a distinct clinical and prognostic subgroup within the AR class.

## Methods

### Study design and inclusion/exclusion criteria

This study was performed in accordance with the STROBE guidelines^30^. Data were collected from a prospective database maintained at the Division of Pancreatic Surgery of San Raffaele Hospital in Milan, Italy. Data collection and all diagnostic and therapeutic procedures were conducted with the signed consent of each patient. Approval from the institution’s ethics committee (number 165/INT/2018) was obtained as part of the VANISSh protocol, registered on ClinicalTrials.gov (NCT04024358).

All consecutive patients with histologically confirmed PDAC undergoing either upfront resection or post-NAT pancreatectomy between January 2015 and December 2022 were included in the study. The following exclusion criteria were applied: locally advanced disease according to National Comprehensive Cancer Network (NCCN) guidelines^31^; patients who underwent R2 resection; patients with distant metastases detected either at diagnosis or intraoperatively; surgery-related deaths (either in-hospital or 90-day postoperative mortality); patients with a follow-up interval of <12 months; and conditional borderline resectable PDAC, defined as those with an Eastern Cooperative Oncology Group (ECOG) performance status ≥2^24^.

### Preoperative workup, classification of resectability, and patient management

All patients underwent standardized pancreatic protocol (arterial and portal phase) chest/abdomen CT at diagnosis, with all images reviewed by two internal expert radiologists (D.P. and F.D.C.) to standardize the classification of all patients according to the latest NCCN criteria. A comprehensive clinical evaluation that focused on the onset of disease-related symptoms (that is abdominal pain, jaundice, weight loss, and acute pancreatitis) was available. For patients with jaundice, CA19-9 levels were assessed after biliary drainage with bilirubin normalization. Patients with jaundice who received upfront surgery underwent biliary drainage before the operation. Endoscopic ultrasonography (EUS) was recommended in all cases and fine needle aspiration with cytological diagnosis was also attempted in patients considered for upfront surgery. MRI or fluorodeoxyglucose-PET (FDG-PET) was performed in selected patients in the case of radiological and/or clinical suspicion of distant or nodal disease. Specifically, in the case of unclear liver findings, MRI with hepatocyte-specific contrast media was performed. All cases were discussed at an internal multidisciplinary meeting. The institutional approach to the management of AR PDAC according to the NCCN was reported in 2009^28^ and 2014^32^. All patients included in the study were classified into two groups based on the resectability status at diagnosis as defined by the latest NCCN criteria, including AR and anatomically borderline resectable (ABR) PDAC. BBR disease was defined as AR cancer, but with adverse prognostic features associated with early recurrence, including: CA19-9 level ≥200 U/ml^26–28^; cancer-related symptoms lasting >40 days (i.e. severe back pain and/or >10% weight loss in the past 3 months^28^); and/or radiological signs of or cytologically proven lymph node metastases^24^. The latter point comprised any suspicion of lymph node involvement, using CT, EUS, MRI, or FDG-PET. All patients meeting the anatomically borderline criteria of the NCCN classification were included in the ABR population. Among them, those who also met the biological criteria were defined as anatomically-biologically borderline resectable (ABBR) patients. All of them, along with BBR PDAC patients, were considered for NAT. Additionally, NAT was offered to some patients with R disease after multidisciplinary evaluation or as part of an ongoing RCT (PACT-21-CASSANDRA (NCT04793932)). In some cases, NAT was not administered because of patient refusal, the referring oncologist’s choice, or patient condition (that is duodenal obstruction with no effective endoscopic palliation, recurrent bleeding due to duodenal infiltration, or advanced age/co-morbidities with no indication for multi-agent chemotherapy). NAT was mainly based on chemotherapy, while radiation was considered only in selected cases. After NAT completion, selection for surgery was based on multidisciplinary board evaluation. Surgical resection was considered on an individual basis in fit patients with potentially R PDAC (patients with pure R PDAC or those eventually requiring a venous vascular resection), showing a decrease in CA19-9 (normalization and/or decrease >90% of baseline value).

### Surgical technique

All operations were performed by the same surgical team using a standard, previously documented, technique^33,34^. To dissect the retropancreatic lamina, identify the origin of the superior mesenteric artery, and promptly detect any vascular involvement, different ‘artery first’ approaches were applied as previously described^33^.

### Data collection

Demographic, clinical, operative, pathological, and postoperative data were recorded. CA19-9 was analysed as a continuous value with four cut-offs of ≥37, ≥200^26–28^, ≥500^22,24^, and ≥1000^24,25^ U/ml. Patients with CA19-9 serum levels ≤5 U/ml were defined as non-secretors. Co-morbidities were classified according to the ASA and the Charlson age co-morbidity index (CACI) classification^35,36^. Tumour node metastasis staging was based on the eighth edition of the AJCC manual^37^. The status of the resection margin was defined as R0 when the distance between tumour cells and the closest resection margin was >1 mm^38^. Tumour regression grade (TRG) was evaluated according to the classification of Hartman and Krasinskas^39^. The recorded postoperative outcomes included severe complications (defined as Clavien–Dindo grade ≥III)^40^ and clinically relevant pancreatic fistula (grade B/C per ISGPS criteria)^41^.

### Postoperative management and follow-up

Adjuvant treatment (chemotherapy/chemoradiation) was always recommended after upfront surgery and based on multidisciplinary evaluation in patients who underwent NAT. Patients who completed 6 months of NAT were eligible for adjuvant chemoradiation if any microscopic margin was positive; patients who completed <6 months of NAT first had to complete the full course of perioperative chemotherapy followed by chemoradiation if needed. Before starting any adjuvant treatment, chest/abdomen CT with contrast and evaluation of serum CA19-9 level were performed within 3 months of surgery. Current institutional follow-up policy and classification of recurrence were described previously^42^. The location of first recurrence was categorized into five mutually exclusive categories: ‘local only’, ‘liver only’, ‘lung only’, ‘multiple sites’, and ‘other’. Multiple-site recurrence included local and distant recurrence and recurrence at multiple distant sites^23^. Peritoneal carcinomatosis was considered in the ‘other’ category (that is patients with only peritoneal recurrence) or the ‘multiple sites’ category. Early recurrence was examined at two time points: within 3^42^ and 12 months after surgery^8^.

### Statistical analysis

Statistical analyses were performed using SPSS^®^ (IBM, Armonk, NY, USA; software package for Macintosh, version 25). Kaplan–Meier curves were drawn using R open software (https://www.r-project.org; version 4.2.3). Categorical variables are presented as *n* (%) and were compared among groups using the chi-squared test or Fisher’s exact test, as appropriate. The distributions of continuous variables are presented as median (interquartile range (i.q.r.)) and were compared using the Mann–Whitney *U* test. Disease-specific survival (DSS) was defined as the interval from the initiation of treatment (either surgery or NAT) to death due to disease or last follow-up. Event-free survival (EFS) was defined as the time from the start of treatment to recurrence, death, or last follow-up. The term ‘event’ was preferred in order to perform an initial analysis considering patients undergoing upfront resection and resection after NAT, as the time of preoperative chemotherapy is not free from disease, but is theoretically an immortal time. Patients who were lost to follow-up or died from other causes were censored. DSS and EFS curves were constructed using the Kaplan–Meier method. Differences between survival probabilities were evaluated using the log rank test. A stepwise multivariable Cox regression analysis with backward elimination was conducted to examine the impact of resectability status on the DSS and EFS, with results presented as HR (95% c.i.). Variables were selected according to literature reports, using the most valuable cut-offs. Factors included in the BBR definition were not included in the univariable and multivariable analysis to avoid collinearity. Variables that exhibited *P* < 0.100 were retained in the final model. Statistical significance was predefined as *P* < 0.050.

## Results

### Baseline cohort characteristics

**Fig. 1:**
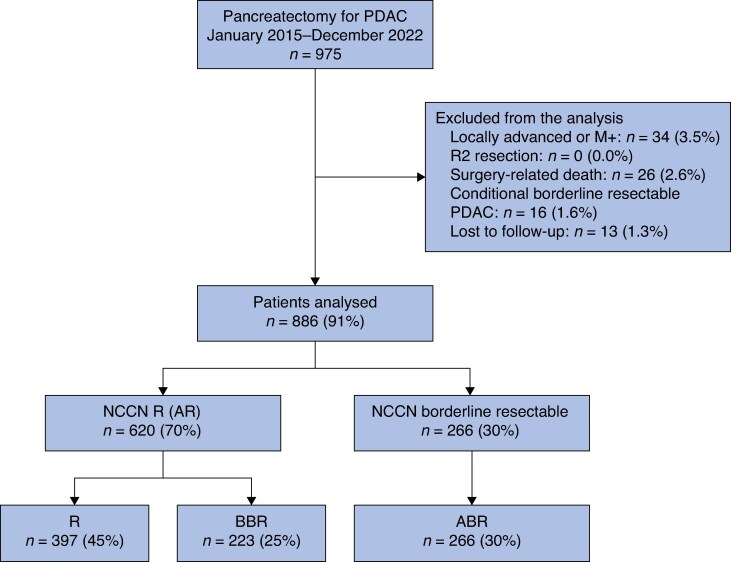
Study flow chart PDAC, pancreatic ductal adenocarcinoma; NCCN, National Comprehensive Cancer Network; R, resectable; AR, anatomically resectable; BBR, biologically borderline resectable; ABR, anatomically borderline resectable.

In the study interval, 975 patients underwent pancreatic resection for histologically proven PDAC. Of these, 886 patients met the inclusion criteria and constitute the study cohort. See *Fig. 1* for the study flow chart. A total of 620 patients (70%) were AR PDAC at diagnosis, of which 397 patients (64%) were R and 223 patients (36%) were BBR. The remaining 266 patients (30%) were ABR. Baseline characteristics of the entire cohort, as well as comparisons between resectability groups, are shown in *Table 1*. Most patients were symptomatic at diagnosis (669 of 886, 75.5%), with jaundice being the most common symptom. Weight loss was more frequent in the BBR group than in the R and ABR groups. The median tumour size at diagnosis in the BBR group (26 (i.q.r. 22–35) mm) was larger than in the R group (23 (i.q.r. 18–30) mm) (*P* < 0.001), but smaller than in the ABR group (30 (i.q.r. 24–35) mm) (*P* = 0.023). At diagnosis, CA19-9 was not expressed in 108 patients (12.2%), without differences between resectability groups. Among secretors, the median CA19-9 level was 85 (i.q.r. 34–250) U/ml and only 52 patients (6.7%) had a CA19-9 level ≥1000 U/ml. The median CA19-9 level at diagnosis in the BBR group was 321 (i.q.r. 116–655) U/ml, significantly higher compared with the R group (46 (i.q.r. 22–88) U/ml) and the ABR group (124 (i.q.r. 47–366) U/ml) (both *P* < 0.001).

**Table 1:** Baseline characteristics of the overall population and in three groups of patients with BBR, R, and ABR pancreatic ductal adenocarcinoma at diagnosis

	Overall (*n* = 886)	BBR (*n* = 223)	R (*n* = 397)	ABR (*n* = 266)	*P*		
					BBR *versus* R	BBR *versus* ABR	R *versus* ABR
Male	454 (51.2)	117 (52.5)	210 (52.9)	139 (52.3)	0.933	0.319	0.205
Age (years), median (i.q.r.)	68 (61.0–73.0)	67 (60.0–73.0)	69 (62.5–75.0)	66 (57.0–71.0)	0.017*	0.011*	<0.001*
BMI (kg/m^2^), median (i.q.r.)	24 (21.8–26.5)	24 (21.7–26.5)	24 (22.0–26.5)	24 (21.6–26.3)	0.880	0.570	0.424
CACI^35^ ≥4	746 (84.2)	186 (83.4)	349 (87.9)	211 (79.3)	0.144	0.296	0.003*
ASA grade ≥III	366 (41.3)	95 (42.6)	158 (39.8)	113 (42.5)	0.497	0.526	0.519
**Symptoms at diagnosis**	669 (75.5)	180 (80.7)	279 (70.3)	210 (78.9)	0.006*	0.653	0.015*
Jaundice	413 (46.6)	111 (49.8)	161 (40.6)	141 (53.0)	0.029*	0.525	0.002*
Pain†	135 (15.2)	39 (17.5)	45 (11.3)	51 (19.2)	0.037*	0.642	0.007*
Acute pancreatitis	63 (7.1)	19 (8.5)	35 (8.8)	9 (3.4)	0.514	0.018*	0.006*
Weight loss	286 (32.3)	90 (40.4)	118 (29.7)	78 (29.3)	0.008*	0.013*	0.931
Tumour size at diagnosis (mm), median (i.q.r.)	25.0 (20.0–32.0)	26.0 (22.0–35.0)	23.0 (18.0–30.0)	30.0 (24.0–35.0)	<0.001*	0.023*	<0.001*
**Tumour site**					0.853	0.014*	0.013*
Head/neck	660 (74.5)	159 (71.3)	286 (72.0)	215 (80.8)			
Body/tail	226 (25.5)	64 (28.7)	111 (28.0)	51 (19.2)			
CA19-9 non-secretors	108 (12.2)	22 (9.9)	54 (13.6)	32 (12.0)	0.202	0.472	0.559
**CA19-9 at diagnosis (U/ml), median (i.q.r.)‡**	85 (34.1–250.0)	321 (116.0–655.0)	46 (22.0–88.0)	124 (47.0–366.0)	<0.001*	<0.001*	<0.001*
≥37	511 (65.7)	158 (78.6)	188 (54.8)	165 (70.5)	<0.001*	0.062	<0.001*
≥200	197 (25.3)	124 (61.6)	0 (0.0)	73 (31.2)	<0.001*	<0.001*	<0.001*
≥500	101 (11.4)	57 (25.6)	0 (0.0)	44 (16.5)	<0.001*	0.010*	<0.001*
≥1000	52 (6.7)	29 (14.4)	0 (0.0)	23 (9.8)	<0.001*	0.182	<0.001*

Values are *n* (%) unless otherwise indicated. *Statistically significant. †Excluded pain due to pancreatitis. ‡Among secretors. BBR, biologically borderline resectable; R, resectable; ABR, anatomically borderline resectable; i.q.r., interquartile range; CACI, Charlson age comorbidity index; CA19-9, carbohydrate antigen 19-9.

### NAT, surgery, and histopathological characteristics

*Table 2* shows NAT details, as well as the surgical, postoperative, and histopathological characteristics of the study cohort and the three groups. NAT was administered in 135 BBR patients (60.5%), 82 R patients (20.7%), and 245 ABR patients (92.1%). The median CA19-9 level before surgery was higher in the BBR group (54 (i.q.r. 26–249) U/ml) than in the R group (39 (i.q.r. 17–88) U/ml) (*P* < 0.001) and the ABR group (28 (i.q.r. 13–58) U/ml) (*P* < 0.001). In keeping with anatomic location and local extension, the BBR group had a lower rate of pancreatoduodenectomy/total pancreatectomy and vascular resections compared with the ABR group.

**Table 2:** Neoadjuvant treatment details and surgical, postoperative, and histopathological characteristics of the overall population and in three groups of patients with BBR, R, and ABR pancreatic ductal adenocarcinoma at diagnosis

	Overall (*n* = 886)	BBR (*n* = 223)	R (*n* = 397)	ABR (*n* = 266)	*P*		
					BBR *versus* R	*BBR versus ABR*	*R versus ABR*
**Neoadjuvant treatment**	462 (52.1)	135 (60.5)	82 (20.7)	245 (92.1)	<0.001*	<0.001*	<0.001*
Chemotherapy	444 (50.1)	126 (56.5)	81 (20.4)	237 (89.1)	<0.001*	<0.001*	<0.001*
Chemoradiation	18 (2.0)	9 (4.0)	1 (0.3)	8 (3.0)			
**Neoadjuvant treatment†**					0.452	0.267	0.531
FOLFIRINOX	190 (41.1)	63 (46.7)	29 (35.4)	98 (40.0)			
Gemcitabine plus nab-paclitaxel	180 (39.0)	44 (32.6)	33 (40.2)	103 (42.0)			
PAXG	65 (14.1)	21 (15.6)	15 (18.3)	29 (11.8)			
Other	27 (5.8)	7 (5.2)	5 (6.1)	15 (6.1)			
CA19-9 before surgery (U/ml), median (i.q.r.)‡	38 (18.0–103.2)	54 (26.0–249.0)	39 (17.5–88.5)	28 (13.0–58.0)	<0.001*	<0.001*	0.008*
**Surgical procedure**					0.972	<0.001*	<0.001*
Pancreatoduodenectomy	591 (66.7)	146 (65.5)	258 (65.0)	187 (70.3)			
Distal pancreatectomy	208 (23.5)	61 (27.4)	108 (27.2)	39 (14.7)			
Total pancreatectomy	87 (9.8)	16 (7.2)	31 (7.8)	40 (15.0)			
Vascular resection	181 (20.4)	28 (12.6)	33 (8.3)	120 (45.1)	0.093	<0.001*	<0.001*
Clavien–Dindo grade ≥III^40^	167 (18.8)	43 (19.3)	70 (17.6)	54 (20.3)	0.665	0.820	0.417
POPF—grade B or C^41^	157 (17.7)	38 (17.1)	82 (20.7)	37 (13.9)	0.427	0.604	0.050
Tumour size at pathology (mm), median (i.q.r.)	25.0 (18.0–30.0)	25.0 (18.0–30.0)	25.0 (18.0–30.0)	25.0 (19.0–30.0)	0.279	0.425	0.821
**T stage (8th edition of the AJCC manual** ^37^ **)**					0.102	0.708	0.453
T0	12 (1.4)	5 (2.2)	3 (0.8)	4 (1.5)			
T1	283 (31.9)	67 (30.0)	137 (34.5)	79 (29.7)			
T2	531 (59.9)	129 (57.8)	238 (59.9)	164 (61.7)			
T3	60 (6.8)	2 (9.9)	19 (4.8)	19 (7.1)			
**N status (8th edition of the AJCC manual** ^37^ **)**					0.357	0.010*	0.031*
N0	268 (30.2)	62 (27.8)	128 (32.2)	78 (29.3)			
N1	337 (38.0)	77 (34.5)	140 (35.3)	120 (45.1)			
N2	281 (31.7)	84 (37.7)	129 (32.5)	68 (25.6)			
**Resection margin** ^38^					0.437	0.271	0.029*
R1 (≤1.0 mm)	354 (40.0)	89 (39.9)	145 (36.5)	120 (45.1)			
Number of harvested lymph nodes, median (i.q.r.)	30.0 (23.0–38.0)	29.0 (23.0–37.0)	28.0 (22.0–36.0)	32.0 (24.0–41.0)	0.495	0.006*	<0.001*
Lymph node ratio, median (i.q.r.)	0.05 (0.00–0.15)	0.07 (0.02–0.19)	0.06 (0.00–0.17)	0.04 (0.00–0.11)	0.183	0.007*	0.052
**Grading**					0.111	0.101	0.873
Poor (G3)	397 (44.8)	112 (50.2)	172 (43.3)	113 (52.5)			
Perineural invasion	691 (78.0)	167 (74.9)	307 (77.3)	217 (81.6)	0.554	0.078	0.206
Lymphovascular invasion	622 (70.2)	156 (70.0)	276 (69.5)	190 (71.4)	0.928	0.765	0.604
**TRG according to Hartman and Kransinskas** ^39^ **†**					0.543	0.229	0.617
Poor	144 (31.1)	37 (27.4)	22 (26.7)	92 (37.5)			
Minimal to moderate	218 (47.2)	63 (47.0)	40 (48.5)	118 (48.2)			
Marked	88 (19.2)	30 (21.9)	17 (21.2)	31 (12.7)			
Complete	12 (2.5)	5 (3.7)	3 (3.6)	4 (1.6)			

Values are *n* (%) unless otherwise indicated. *Statistically significant. †Referring only to patients undergoing neoadjuvant treatment. ‡Among secretors. BBR, biologically borderline resectable; R, resectable; ABR, anatomically borderline resectable; FOLFIRINOX, fluorouracil, leucovorin, irinotecan, and oxaliplatin; PAXG, nab-paclitaxel plus gemcitabine, capecitabine, and cisplatin; CA19-9, carbohydrate antigen 19-9; i.q.r., interquartile range; POPF, postoperative pancreatic fistula; TRG, tumour regression grade.

The lymph node ratio (LNR) was similar between BBR and R patients (0.07 (i.q.r. 0.02–0.19) *versus* 0.06 (i.q.r. 0.00–0.17) respectively, *P* = 0.183), whereas the rate of pN2 patients was higher in the BBR group than in the R group (37.7% *versus* 32.5% respectively) although this difference was not statistically significant (*P* = 0.357). BBR patients had a higher rate of pN2 tumours than ABR patients (37.7% *versus* 25.6% respectively, *P* = 0.001) and a higher median LNR than ABR patients (0.07 (i.q.r. 0.0–0.2) *versus* 0.04 (i.q.r. 0.0–0.1) respectively, *P* = 0.007). R patients had a more favourable pathological profile, with a statistically significant higher rate of negative nodes compared with ABR patients (32.2% *versus* 29.3% respectively, *P* = 0.031) and a statistically significant higher rate of R0 resection compared with ABR patients (63.5% *versus* 54.9% respectively, *P* = 0.029).

### Adjuvant treatment and recurrence

*Table 3* shows adjuvant treatment details, recurrence details, and survival outcomes.

**Table 3:** Adjuvant treatment details, recurrence details, and survival outcomes of the overall population and in three groups of patients with BBR, R, and ABR pancreatic ductal adenocarcinoma at diagnosis

	Overall (*n* = 886)	BBR (*n* = 223)	R (*n* = 397)	ABR (*n* = 266)	*P*		
					BBR *versus* R	BBR *versus* ABR	R *versus* ABR
**Adjuvant treatment**	632 (71.3)	157 (70.4)	296 (74.6)	179 (67.3)	0.299	0.494	0.044*
Chemotherapy alone	380 (42.9)	96 (43.0)	208 (52.4)	76 (28.6)	0.077	0.006*	<0.001*
Chemoradiation	223 (25.2)	53 (23.8)	82 (20.7)	88 (33.1)			
Radiation alone	29 (3.3)	8 (3.6)	6 (1.5)	15 (5.6)			
**Adjuvant chemotherapy regimen**					0.123	0.441	0.433
Gemcitabine	286 (32.3)	70 (31.4)	160 (40.3)	56 (38.3)			
FOLFIRINOX	104 (11.7)	22 (9.9)	51 (12.8)	31 (11.7)			
Capecitabine	66 (7.4)	22 (9.9)	18 (4.5)	26 (9.8)			
Gemcitabine plus nab-paclitaxel	76 (8.6)	18 (8.1)	31 (7.8)	27 (10.2)			
Gemcitabine plus capecitabine	21 (2.4)	4 (1.8)	13 (3.3)	4 (1.5)			
PAXG	24 (2.7)	4 (1.8)	10 (2.5)	10 (3.8)			
Other	26 (2.9)	9 (4.0)	7 (1.8)	10 (3.8)			
**First recurrence type**					0.176	0.440	0.099
Local only	101 (11.4)	21 (9.4)	43 (10.8)	37 (13.9)			
Liver only	158 (17.8)	49 (22.0)	50 (12.6)	59 (22.2)			
Lung only	93 (10.5)	24 (10.8)	46 (11.6)	23 (8.6)			
Multiple sites	109 (12.3)	34 (15.2)	43 (10.8)	32 (12.0)			
Other	109 (12.3)	32 (14.3)	44 (11.1)	33 (12.4)			
Very early recurrence (≤3 months)	76 (8.7)	26 (11.7)	26 (6.5)	24 (9.0)	0.034*	0.370	0.237
Early recurrence (≤12 months)	324 (36.5)	110 (49.3)	96 (24.1)	118 (44.3)	<0.001*	0.277	<0.001*
**Survival (months), median (95% c.i.)**							
Disease-specific survival	44 (40.6,47.3)	40 (30.0,49.9)	59 (51.8,66.1)	40 (36.0,44.9)	<0.001*	0.570	0.001*
Event-free survival	23 (20.8,25.1)	19 (17.2,20.7)	29 (23.9,33.5)	23 (20.2,25.7)	<0.001*	0.051	0.012*

Values are *n* (%) unless otherwise indicated. *Statistically significant. BBR, biologically borderline resectable; R, resectable; ABR, anatomically borderline resectable; FOLFIRINOX, fluorouracil, leucovorin, irinotecan, and oxaliplatin; PAXG, nab-paclitaxel plus gemcitabine, capecitabine, and cisplatin.

Adjuvant chemotherapy was administered in 632 of all patients (71.3%) and the majority (380 of 632, 60.1%) received chemotherapy alone. The rate of adjuvant treatment receipt did not differ between the BBR group and the R group (70.4% *versus* 74.6% respectively; *P* = 0.299).

During the follow-up interval, tumour recurrence was diagnosed in 570 patients (64.3%) and 324 of these (56.8%) experienced the tumour recurrence within 12 months after surgery. The BBR group showed a higher rate of early recurrence within 12 months after surgery than the R group (49.3% *versus* 24.1% respectively, *P* < 0.001), but a similar rate to the ABR group (49.3% *versus* 44.3% respectively, *P* = 0.277). Most patients (469 of 886, 52.9%) exhibited a systemic recurrence, whereas 11.4% (101 of 886) had isolated local relapse. The recurrence pattern did not differ significantly among the resectability groups.

### Survival analyses

**Fig. 2:**
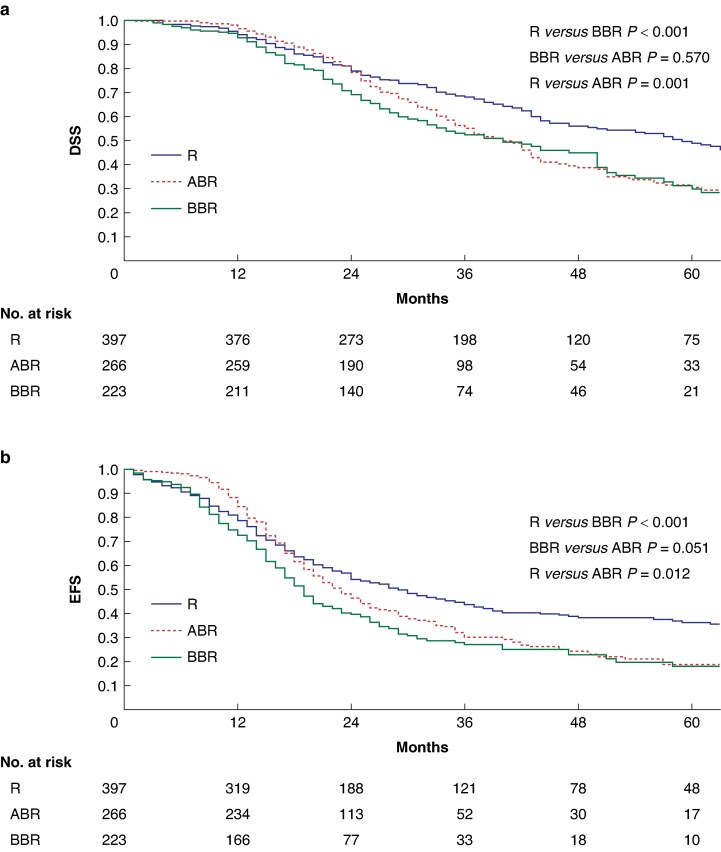
Comparison of survival among R, BBR, and ABR patients **a** DSS. **b** EFS. R, resectable; BBR, biologically borderline resectable; ABR, anatomically borderline resectable; DSS, disease-specific survival; EFS, event-free survival.

After a median follow-up of 45 (95% c.i. 42 to 48) months, the median DSS and EFS of the entire cohort were 44 (95% c.i. 40 to 47) and 23 (95% c.i. 20 to 25) months respectively. The median DSS of the BBR, R, and ABR groups was 40 (95% c.i. 30 to 49), 59 (95% c.i. 51 to 66), and 40 (95% c.i. 36 to 44) months respectively. The 1- and 3-year DSS estimates in the BBR group (92% and 52% respectively) were significantly lower than those in the R group (94% and 68% respectively) (*P* < 0.001), whereas no significant difference was observed compared with the ABR group (98% and 66% respectively) (*P* = 0.570)(*Fig. 2a*). The median EFS in the BBR group was 19 (95% c.i. 17 to 20) months, significantly shorter compared with the R group (29 (95% c.i. 23 to 33) months), but comparable to the ABR group (23 (95% c.i. 20 to 25) months) (*Fig. 2b*). The 1- and 3-year EFS estimates were 72% and 27% respectively in the BBR group *versus* 78% and 43% respectively in the R group (*P* < 0.001).

### Multivariable analyses

Multivariable analysis (*Table 4*) showed that age (HR 1.01 (95% c.i. 1.01 to 1.02), *P* = 0.027), tumour size at diagnosis (HR 1.01 (95% c.i. 1.00 to 1.02), *P* < 0.001), ABR (HR 2.60, 95% c.i. 1.93 to 3.51, *P* < 0.001), and BBR (HR 2.09 (95% c.i. 1.61 to 2.70), *P* < 0.001) were independently associated with shorter DSS. Tumour size at diagnosis (HR 1.01 (95% c.i. 1.00 to 1.02), *P* < 0.001), ABR (HR 1.70 (95% c.i. 1.32 to 2.19), *P* < 0.001), and BBR (HR 1.80 (95% c.i. 1.44 to 2.25), *P* < 0.001) were also independently associated with shorter EFS. Conversely, NAT was independently associated with longer DSS (HR 0.44 (95% c.i. 0.34 to 0.57), *P* < 0.001) and EFS (HR 0.57 (95% c.i. 0.46 to 0.71), *P* < 0.001).

**Table 4:** Univariable and multivariable analysis of factors associated with DSS and EFS in the whole study cohort (*n* = 886)

	DSS				EFS			
	Univariable analysis		Multivariable analysis		Univariable analysis		Multivariable analysis	
	HR (95% c.i.)	*P*	HR (95% c.i.)	*P*	HR (95% c.i.)	*P*	HR (95% c.i.)	*P*
Female	1.02 (0.85,1.24)	0.781			0.94 (0.79,1.11)	0.470		
Age	1.01 (1.00,1.02)	0.026*	1.01 (1.01,1.02)	0.027*	0.99 (0.98,1.00)	0.323		
ASA grade ≥III	1.32 (1.09,1.60)	0.004*	1.19 (0.98,1.44)	0.067	1.07 (0.90,1.26)	0.403		
CA19-9 9 at diagnosis	1.00 (1.00,1.00)	0.520			1.00 (1.00,1.00)	0.610		
Tumour size at diagnosis	1.01 (1.00,1.01)	0.003*	1.01 (1.00,1.02)	<0.001*	1.01 (1.00,1.02)	<0.001*	1.01 (1.00,1.02)	<0.001*
Neoadjuvant treatment	0.79 (0.65,0.95)	0.016*	0.44 (0.34,0.57)	<0.001*	0.86 (0.73,1.02)	0.094	0.57 (0.46,0.71)	<0.001*
Body/tail *versus* head/neck lesion	0.90 (0.72,1.13)	0.389			1.03 (0.85,1.25)	0.716		
**Resectability status**								
R	1		1		1		1	
ABR	1.44 (1.15,1.80)	0.001*	2.60 (1.93,3.51)	<0.001*	1.27 (1.05,1.55)	0.014*	1.70 (1.32,2.19)	<0.001*
BBR	1.54 (1.22,1.95)	<0.001*	2.09 (1.61,2.70)	<0.001*	1.55 (1.26,1.90)	<0.001*	1.80 (1.44,2.25)	<0.001*

*Statistically significant. DSS, disease-specific survival; EFS, event-free survival; CA19-9, carbohydrate antigen 19-9; R, resectable; ABR, anatomically borderline resectable; BBR, biologically borderline resectable.

### Subgroup analyses

**Fig. 3:**
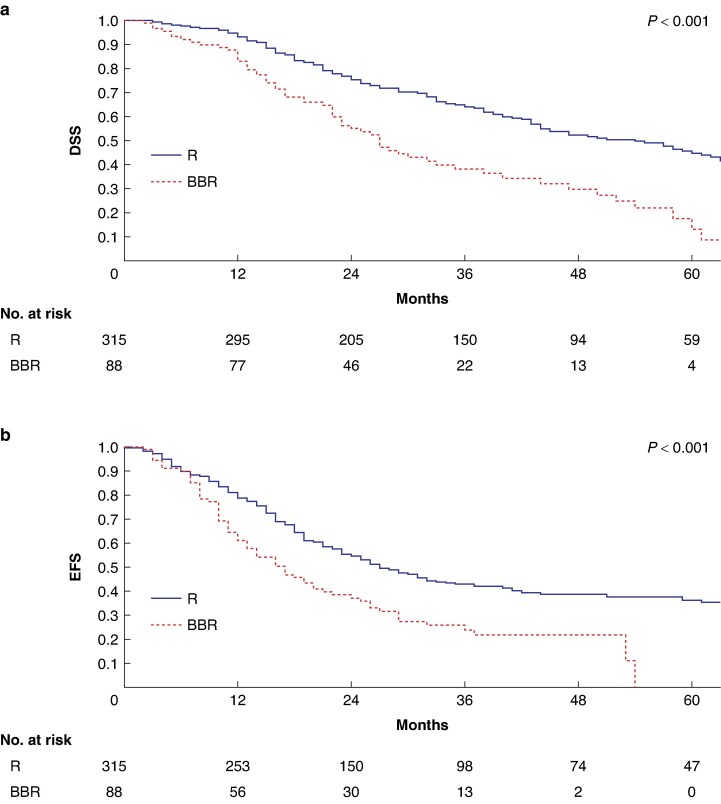
Comparison of survival among R and BBR patients who underwent upfront surgery **a** DSS. **b** EFS. R, resectable; BBR, biologically borderline resectable; DSS, disease-specific survival; EFS, event-free survival.

*Table 5* shows the comparison between patients who underwent upfront surgery in the R group (315 of 397, 79.3%) and the BBR group (88 of 223, 39.4%). BBR patients had a less favourable pathological profile compared with the R group, in terms of larger median tumour size at final pathology (28 *versus* 25 mm respectively, *P* < 0.001), higher pT stage (pT2–pT3: 84.1% *versus* 67.6% respectively, *P* = 0.025), more pN2 tumours (55.7% *versus* 35.9% respectively, *P* < 0.001), and higher median LNR (0.16 *versus* 0.07 respectively, *P* < 0.001). Among these subgroups, 1- and 3-year DSS estimates were 83% and 38% respectively in the BBR group (median of 27 (i.q.r. 21–32) months) compared with 93% and 63% respectively in the R group (median of 54 (i.q.r. 44–63) months) (*P* < 0.001) (*Fig. 3a*). The 1- and 3-year EFS estimates in upfront resected BBR patients (51% and 22% respectively; median of 13 (i.q.r. 9–16) months) were significantly lower than in upfront resected R patients (74% and 42% respectively; median of 24 (i.q.r. 19–28) months) (*P* < 0.001) (*Fig. 3b*). Multivariable analysis in upfront resected R and BBR patients (*Table S1*) showed that tumour size at diagnosis and BBR were independently associated with DSS (HR 1.02 (95% c.i. 1.00 to 1.03) (*P* = 0.002) and 1.94 (95% c.i. 1.36 to 2.76) (*P* < 0.001) respectively) and EFS (HR 1.01 (95% c.i. 1.00 to 1.03) (*P* < 0.001) and 1.43 (95% c.i. 1.00 to 2.06) (*P* = 0.004) respectively).

**Table 5:** Demographics and clinical, treatment, and histopathological characteristics of upfront resected BBR and R pancreatic ductal adenocarcinoma at diagnosis

	BBR (*n* = 88)	R (*n* = 315)	*P*
Male	49 (55.7)	162 (51.4)	0.546
Age (years), median (i.q.r.)	71 (64.0–77.0)	70 (63.0–75.0)	0.304
BMI (kg/m^2^), median (i.q.r.)	24 (21.9–26.4)	24 (22.0–26.4)	0.804
CACI^35^ ≥4	79 (89.8)	277 (87.9)	0.711
ASA grade ≥III	45 (51.5)	126 (40.0)	0.068
**Symptoms at diagnosis**	80 (90.9)	223 (70.8)	<0.001*
Jaundice	56 (63.6)	135 (42.9)	0.001*
Pain†	17 (19.3)	35 (11.1)	0.049*
Acute pancreatitis	7 (8.0)	29 (9.2)	0.834
Weight loss	50 (56.8)	99 (31.4)	<0.001*
Tumour size at diagnosis (mm), median (i.q.r.)	25.0 (20.0–30.0)	22.0 (18.0–30.0)	0.047*
**Tumour site**			0.160
Head/neck	72 (81.8)	234 (74.3)	
Body/tail	16 (18.2)	81 (25.7)	
CA19-9 non-secretors	5 (5.7)	44 (14.0)	0.055
**CA19-9 at diagnosis (U/ml), median (i.q.r.)‡**	300 (80.0–492.0)	43 (20.0–89.0)	<0.001*
≥37	56 (67.5)	144 (53.1)	0.023*
≥200	48 (57.8)	0 (0.0)	<0.001*
≥500	16 (18.2)	0 (0.0)	<0.001*
≥1000	4 (4.8)	0 (0.0)	<0.001*
**Surgical procedure**			0.100
Pancreatoduodenectomy	69 (78.4)	211 (67.0)	
Distal pancreatectomy	13 (14.8)	79 (25.1)	
Total pancreatectomy	6 (6.8)	25 (7.9)	
Vascular resection	11 (12.5)	31 (9.8)	0.553
Clavien–Dindo grade ≥III^40^	20 (22.7)	55 (17.5)	0.279
POPF—grade B or C^41^	17 (19.3)	68 (21.6)	0.891
Tumour size at pathology (mm), median (i.q.r.)	28.0 (23.0, 35.0)	25.0 (19.0, 30.0)	<0.001*
**T stage (8th edition of the AJCC manual** ^37^ **)**			0.025*
T1	14 (15.9)	102 (32.4)	
T2	66 (75.0)	197 (62.5)	
T3	8 (9.1)	16 (5.1)	
**N status (8th edition of the AJCC manual** ^37^ **)**			<0.001*
N0	10 (11.4)	94 (29.8)	
N1	29 (33.0)	108 (34.3)	
N2	49 (55.7)	113 (35.9)	
**Resection margin** ^38^			0.222
R1 (≤1.0 mm)	42 (47.7)	126 (40.0)	
Number of harvested lymph nodes, median (i.q.r.)	27.0 (23.2–34.0)	28.0 (21.0–36.0)	0.819
Lymph node ratio, median (i.q.r.)	0.16 (0.05–0.28)	0.07 (0.00–0.19)	<0.001*
**Grading**			0.016*
Poor (G3)	52 (59.1)	140 (44.4)	
Perineural invasion	71 (80.7)	249 (79.0)	0.768
Lymphovascular invasion	76 (86.4)	227 (72.1)	0.008*
**Adjuvant treatment**	72 (81.8)	244 (77.5)	0.464
Chemotherapy alone	53 (60.2)	183 (58.1)	0.214
Chemoradiation	15 (17.0)	57 (18.1)	
Radiation alone	4 (4.5)	4 (1.3)	
**Adjuvant chemotherapy regimen**			0.618
Gemcitabine	47 (53.4)	153 (48.6)	
FOLFIRINOX	6 (6.8)	37 (11.7)	
Capecitabine	4 (4.5)	8 (2.5)	
Gemcitabine plus nab-paclitaxel	5 (5.7)	20 (6.3)	
Gemcitabine plus capecitabine	3 (3.4)	12 (3.8)	
PAXG	2 (2.3)	5 (1.6)	
Other	1 (1.1)	5 (1.6)	
**First recurrence type**	66 (75.0)	185 (58.7)	0.006*
Local only	11 (12.5)	37 (11.7)	
Liver only	18 (20.5)	40 (12.7)	
Lung only	7 (8.0)	31 (9.8)	
Multiple sites	17 (19.3)	41 (13.0)	
Other	13 (14.8)	36 (11.4)	
Very early recurrence (≤3 months)	10 (11.3)	21 (6.6)	0.273
Early recurrence (≤12 months)	42 (47.7)	81 (25.7)	0.003*
**Survival (months), median (95% c.i.)**			
DSS	27 (21.5,32.4)	54 (44.9,63.0)	<0.001*
EFS	13 (9.7,16.2)	24 (19.1,28.8)	<0.001*

Values are *n* (%) unless otherwise indicated. *Statistically significant. †Excluded pain due to pancreatitis. ‡Among secretors. BBR, biologically borderline resectable; R, resectable; i.q.r., interquartile range; CACI, Charlson age co-morbidity index; CA19-9, carbohydrate antigen 19-9; POPF, postoperative pancreatic fistula; FOLFIRINOX, fluorouracil, leucovorin, irinotecan, and oxaliplatin; PAXG, nab-paclitaxel plus gemcitabine, capecitabine, and cisplatin; DSS, disease-specific survival; EFS, event-free survival.

See *Fig. S1* for the comparison of DSS and EFS between the subgroups of R (82 of 397, 20.7%) and BBR (135 of 223, 60.5%) patients who underwent surgical resection after NAT. The median DSS in these groups was 76 (95% c.i. 59 to 92) months for R patients compared with 50 (95% c.i. 43 to 56) months for BBR patients. The 1- and 3-year DSS estimates were 98% and 83% respectively in the R group *versus* 99% and 62% respectively in the BBR group (*P* = 0006). Among these subgroups, the 1- and 3-year EFS estimates for BBR patients (85% and 30% respectively; median of 20 (i.q.r. 23–32) months) were significantly lower than for R patients (96% and 83% respectively; median of 38 (i.q.r. 28–47) months) (*P* < 0.001).

See *Fig. S2* for the comparison of DSS and EFS between the subgroups of BBR patients who underwent upfront resection (88 of 223, 39.5%) and those resected after NAT (135 of 223, 60.5%). The median DSS was 27 (95% c.i. 21 to 32) months and 50 (95% c.i. 43 to 56) months in the upfront BBR patients and the NAT BBR patients respectively (*P* < 0.001). Among these subgroups, the 1- and 3-year EFS estimates for the upfront BBR patients (57% and 22% respectively; median of 20 (i.q.r. 17–22) months) were significantly lower than for the NAT BBR patients (85% and 30% respectively; median of 19 (i.q.r. 17–20) months) (*P* = 0.001).

Within the ABR group, 110 patients (41.3%) also met the biological criteria (ABBR). *Table S2* shows the comparison between the ABR without biological criteria (156 of 266, 58.7%) and ABBR (110 of 266, 41.3%) subgroups, revealing overall homogeneity without significant differences.

## Discussion

AR PDAC is a highly variable category with conflicting survival rates. In this retrospective study based on a prospectively collected series, an institutional BBR population was analysed, with patients selected based on the AR population according to the NCCN^31^, but with specific clinical, radiological, and tumour marker features that are associated with more aggressive disease and therefore with an increased risk of cancer recurrence and worse survival^8,22–24^. Based on previous literature, three criteria were applied: CA19-9 levels ≥200 U/ml^27,28^; cancer-related symptoms lasting >40 days^28^; and/or radiological suspicion of or cytologically proven lymph node metastases at diagnosis^24^. Overall, these criteria led to the selection of patients with worse DSS and EFS within the AR population, particularly in the patients treated with upfront surgical resection. Focusing on CA19-9, a cut-off ≥200 U/ml was considered, as it has been associated with a higher risk of cancer recurrence^26^, cancer-related mortality within 1 year after resection^28^, and a higher rate of occult metastases at surgical exploration^27^. However, nowadays, the most commonly applied threshold for CA19-9 is >500, as it was the first to be proposed by the consensus conference in 2017^24^ and was recently used in a study by Dekker *et al*.^22^. Specifically, Dekker *et al*.^22^ applied a simplified scoring system in a cohort of patients, with all NCCN anatomical classes treated with FOLFIRINOX, adding one point for each independent predictor of poorer survival, including baseline CA19-9 >500 U/ml, performance status ≥1, and ABR PDAC, and adding two points for locally advanced disease. This scoring system had a good prognostic performance for overall survival, with CA19-9 >500 U/ml identified as an independent predictor of worse OS, yielding an HR of 1.36 (95% c.i. 1.21 to 1.52).

Nodal involvement is a well-established prognostic factor in resected PDAC^3,8,11,43,44^, such that preoperative evidence of nodal involvement should be considered a sign of more aggressive disease biology. The presence of cancer-related symptoms lasting >40 days was initially proposed by Barugola *et al*.^28^ as a predictor of worse 1-year survival after PDAC resection, particularly referring to persistent abdominal/back pain. Of note, pain is related to perineural invasion, which is a crucial predictor of DSS^45–48^. However, this factor is the weakest in the present study, as it is not widely adopted and exhibits significant variability, relying heavily on clinical evaluation.

Of 886 patients included in the study, 620 (70%) were AR, of whom 223 (36%) were BBR and 397 (64%) were R. Patients with BBR disease had a worse nodal pattern compared with those with ABR disease, with a higher rate of pN2 and a greater LNR. This difference in nodal pattern was not observed when comparing R and BBR patients, even though the latter group included an approximately three-fold higher proportion of patients undergoing NAT. However, when the analysis was restricted to upfront resected patients, BBR patients had a worse pathological profile than R patients.

The median estimated DSS and EFS of BBR patients were 40 and 19 months respectively. These were significantly shorter compared with those of R patients (59 and 29 months respectively), whereas no significant differences were observed when compared with ABR patients (40 and 23 months respectively). Within the BBR group, patients who underwent upfront surgery had a significantly worse DSS (27 *versus* 50 months) and EFS (19 *versus* 20 months) compared with those receiving post-neoadjuvant pancreatectomy. When restricting the analysis to patients who underwent resection after NAT, BBR patients still had a worse prognosis than R patients, with shorter DSS (50 *versus* 76 months respectively) and EFS (20 *versus* 38 months respectively). This result gains even more relevance considering that the BBR cohort is enriched in patients who did not progress during treatment and remained fit enough for surgical resection.

The findings of this study indicate that, despite sharing anatomical similarities, BBR disease and R disease represent different prognostic entities and should be classified as distinct diseases. Conversely, BBR disease and ABR disease exhibit considerable similarities. Approximately half of the patients in this study underwent NAT, with significant differences between the three groups (92.1% in the ABR group, 60.5% in the BBR group, and 20.7% in the R group). To mitigate the influence of NAT on the comparison between R and BBR patients, a separate analysis was conducted, focusing on the subgroup of AR PDAC patients treated with upfront surgery. In this cohort, which the authors believe is more representative of the natural history of the disease, the median DSS and EFS of R patients were significantly longer than those of BBR patients (54 *versus* 27 months and 24 *versus* 13 months respectively). All these data underline that BBR patients have a poorer prognosis than other AR patients, even if they share the same radiological aspect. This finding was further supported by the results of a multivariable analysis, which demonstrated that the BBR criteria were independently associated with worse DSS and EFS, with HRs of 1.94 and 1.43 respectively. The combination of criteria used to define a BBR cancer appears to be more significant than CA19-9 values alone, as well as other covariates examined at diagnosis. Other independent predictors of worse DSS and EFS were tumour size at diagnosis and age, whereas NAT was independently associated with better survival outcomes. These results are consistent with the TAPS Consortium study^22^ and underscore that several features beyond ‘anatomy’ (that is performance status, CA19-9 values, symptoms, radiological tumour size, and lymph node status) should be considered when evaluating patients with localized pancreatic cancer.

The present study has several limitations pertaining to its retrospective nature. The analysis of individuals undergoing NAT may be subject to selection bias, mostly because of the diversity in indications for NAT and in the type and duration of chemotherapy regimens. Furthermore, the initial number of patients who started NAT, but did not proceed to surgery, is also unavailable. Thus, generalization of these results to patients who did not receive resection is not possible. This aspect is particularly important for the ABR group, where the rate of patients who undergo surgery after NAT according to literature reports may be lower^13^, precluding comparisons between this group and the others. In addition, the number of BBR patients who underwent upfront surgery is quite small, as the authors routinely recommended NAT for all these individuals. It is also possible that the selection criteria that have been adopted for surgery after NAT may have resulted in the exclusion of certain patients. Moreover, BBR criteria were arbitrarily established based on previous research, potentially overlooking other significant elements. Finally, BBR status was recorded prospectively, but without collecting each individual variable that composes it separately, meaning that the specific impact of its distinct parameters could not be ascertained.

The findings of this study indicate the existence of a subset of patients (BBR) with AR pancreatic cancer, as defined by NCCN^31^ criteria, who exhibit a distinctive prognosis. This group necessitates separate evaluation during studies and could warrant distinct treatment, which may comprise NAT. Further studies to validate these criteria and estimate the most proper treatment, ideally through a prospective approach, are required.

## Supplementary Material

zraf033_Supplementary_Data

## Data Availability

The data that support the findings of this study are available from the corresponding author (S.C.), upon reasonable request.
