# Supplementary material for: Anatomically resectable versus biologically borderline resectable pancreatic cancer definition: refining the border beyond anatomical criteria and biological aggressiveness
Source: BJS Open. 2025 May 20;9(3):zraf033. doi: 10.1093/bjsopen/zraf033 (PMC12090895; doi:10.1093/bjsopen/zraf033)
Supplement: zraf033_Supplementary_Data [file zraf033_supplementary_data.docx]

**ANATOMICALLY RESECTABLE VERSUS BIOLOGICAL BORDERLINE RESECTABLE PANCREATIC CANCER DEFINITION: REFINING THE BORDER BEYOND ANATOMICAL CRITERIA AND BIOLOGICAL AGGRESSIVENESS**

**Authors**: Giulio Belfiori MD^1^*, Federico De Stefano MD^1,2^*, Domenico Tamburrino MD, PhD^1^, Giulia Gasparini MD, PhD^1,2^, Francesca Aleotti MD^1^, Paolo Riccardo Camisa MD^1,2^, Claudia Arcangeli MD^1,2^, Marco Schiavo Lena MD^3^, Nicolo Pecorelli MD^1,2^, Diego Palumbo MD, PhD^4^, Stefano Partelli MD^1,2^, Francesco De Cobelli MD^4,2^, Michele Reni MD^5,2^, Stefano Crippa MD, PhD^1,2^ and Massimo Falconi MD^1,2^

**Affiliation**: ^1^Division of Pancreatic Surgery, Pancreas Translational & Clinical Research Center, IRCCS San Raffaele Scientific Institute, Milan, Italy; ^2^Vita-Salute San Raffaele University, Milan, Italy, ^3^Department of Pathology, IRCCS San Raffaele Scientific Institute, Milan, Italy; ^4^Department of Radiology, IRCCS San Raffaele Scientific Institute, Milan, Italy; ^5^Department of Oncology, IRCCS San Raffaele Scientific Institute, Milan, Italy

* Giulio Belfiori and Federico De Stefano share the first authorship

**Corresponding author**:

Stefano Crippa, MD, PhD

Department of Surgery, Division of Pancreatic Surgery

San Raffaele Scientific Institute, Via Olgettina 60, 20132 Milan – Italy

Phone: +39-02-2643.3279/6591

Email: [crippa.stefano@hsr.it](mailto:crippa.stefano@hsr.it) **ORCID ID**; [0000-0002-7370-1508](http://orcid.org/0000-0002-7370-1508)  **Twitter**  @Stefanocrippa6

**Original Article**

**Funding:**

Stefano Crippa received a grant from Fondazione Nadia Valsecchi. This work was supported by a Research Grant from the Italian Association for Cancer Research (AIRC) – AIRC Special program in Metastatic Disease: the key unmet need in oncology, 5 per Mille no. 22737 to Massimo Falconi and Michele Reni.

**The data that support the findings of this study are available from the corresponding author, [S.C.], upon reasonable request.**

**Supplementary Materials - Index**

| **Supplementary Tables** |  |
| --- | --- |
| Supplementary Table 1 | *pag. 3* |
| Supplementary Table 2 | *pag. 4* |
| **Supplementary Figures** |  |
| Supplementary Figure 1 | *pag. 6* |
| Supplementary Figure 2 | *pag. 6* |
|  |  |

**Supplementary Tables**

**Supplementary Table 1.** Univariable and multivariable analysis of factors associated with disease-specific survival (DSS) and event-free survival (EFS) in patients with resectable (R) and biological borderline resectable (BBR) pancreatic ductal adenocarcinoma at diagnosis treated with upfront resection (*n*= 403).

|  | **Disease-Specific Survival (DSS)** | | | | | **Event-Free Survival (EFS)** | | | |
| --- | --- | --- | --- | --- | --- | --- | --- | --- | --- |
|  | **Univariable analysis** | | **Multivariable analysis** | | | **Univariable analysis** | | **Multivariable analysis** | |
|  | HR  [95%CI] | *P-value* | HR  [95%CI] | | *P-value* | HR  [95%CI] | *P-value* | HR  [95%CI] | *P-*  *value* |
| Gender: Female | 0.93  [0.71 – 1.22] | 0.628 |  |  | | 0.92  [0.71 – 1.18] | 0.514 |  |  |
| Age | 1.01  [0.99 – 1.03] | 0.071 | 1.00  [0.98 – 1.02] | 0.427 | | 1.00  [0.98 – 1.01] | 0.821 |  |  |
| ASA ≥ 3 | 1.42  [1.08 – 1.87] | **0.012** | 1.31  [0.96 – 1.78] | 0.089 | | 1.26  [0.98 – 1.62] | 0.067 | 1.22  [0.92 – 1.62] | 0.167 |
| CA19-9 9 at diagnosis | 1.00  [1.00 – 1.01] | **0.003** | 1.00  [1.00 – 1.00] | 0.415 | | 1.00  [1.00 – 1.00] | **< 0.001** | 1.00  [1.00 – 1.00] | 0.055 |
| Tumor size at diagnosis | 1.02  [1.00 – 1.03] | **< 0.001** | 1.02  [1.00 – 1.03] | **0.002** | | 1.02  [1.01 – 1.03] | **<0.001** | 1.01  [1.00 – 1.03] | **<0.001** |
| Body-Tail vs.  Head-Neck Lesion | 0.95  [0.68 – 1.32] | 0.770 |  |  | | 0.88  [0.65 – 1.19] | 0.418 |  |  |
| **Resectability Status**  - Resectable  - BBR | 1  2.24  [1.65 – 3.03] | **< 0.001** | 1  1.94  [1.36 – 2.76] | **<0.001** | | 1  1.78  [1.34 – 2.37] | **< 0.001** | 1  1.43  [1.00 – 2.06] | **0.004** |
| *BBR = biological borderline resectable.* | | | | | | | | | |

**Supplementary Table 2**. Demographics, clinical, treatment and histopathological characteristics of patients with anatomical borderline-resectable disease that also met the biological-borderline criteria (ABBR) and anatomical borderline-resectable patients that did not (ABR without biological criteria) at diagnosis.

|  | | **ABBR** | **ABR without biological criteria** | *P* Value |
| --- | --- | --- | --- | --- |
| n | | 110 | 156 |  |
| Gender male | | 54 (49.1) | 85 (54.5) | 0.455 |
| Age (years) | | 66 [57.0, 71.2] | 66 [58.0, 70.0] | 0.454 |
| BMI (kg/m^2^) | | 23 [21.3, 26.2] | 24 [21.7, 26.3] | 0.244 |
| CACI^35^ ≥ 4 | | 89 (80.9) | 122 (78.2) | 0.646 |
| ASA ≥ 3 | | 47 (42.7) | 66 (42.3) | 0.523 |
| Symptoms at diagnosis  Jaundice  Pain^*^  Acute pancreatitis  Weight loss | | 87 (79.1)  61 (55.5)  26 (23.6)  3 (2.7)  35 (31.8) | 123 (78.8)  80 (51.3)  25 (16.0)  6 (3.8)  43 (27.6) | 0.544  0.534  0.154  0.740  0.495 |
| Tumor size at diagnosis (mm) | | 30.0 [25.0, 40.0] | 29.0 [23.0, 35.0] | 0.050 |
| Tumor site  Head/Neck  Body/Tail | | 89 (80.9)  21 (19.1) | 126 (80.8)  30 (19.2) | 0.554 |
| CA19-9 non secretors | | 7 (6.4) | 25 (16.0) | **0.021** |
| CA19-9 at diagnosis^§^ | | 395 [200.0, 955.0] | 64 [30.0, 115.0] | **<0.001** |
| Neoadjuvant treatment  Chemotherapy  Chemoradiation | | 99 (90.0)  95 (86.4)  4 (3.6) | 146 (93.6)  142 (91.0)  4 (2.6) | 0.357 |
| Neoadjuvant treatment regimen^**^  FOLFIRINOX  Gem-Abraxane  PAXG  Other | | 41 (41.4)  43 (43.4)  8 (8.1)  7 (7.1) | 57 (39.0)  60 (41.1)  21 (14.4)  8 (5.5) | 0.504 |
| CA19-9 before surgery^§^ | | 44 [20.0, 123.0] | 29 [23.0 , 35.0] | **<0.001** |
| Surgical Procedure  Pancreaticoduodenectomy  Distal Pancreatectomy  Total pancreatectomy | | 79 (71.8)  16 (14.5)  15 (13.6) | 108 (69.2)  23 (14.7)  25 (16.0) | 0.872 |
| Vascular resection | | 48 (43.6) | 72 (46.2) | 0.709 |
| Clavien-Dindo ≥3^40^ | | 16 (14.5) | 38 (24.4) | 0.063 |
| POPF - grade B or C^41^ | | 14 (12.7) | 23 (14.8) | 0.436 |
| Tumor size at pathology (mm) | | 25.0 [20.0, 30.0] | 24.0 [18.0, 29.7] | 0.347 |
| T-Stage AJCC 8^th37^  T0  T1  T2  T3 | | 2 (1.8)  28 (25.4)  69 (62.7)  11 (10.0) | 2 (1.3)  51 (32.7)  95 (60.9)  8 (5.1) | 0.447 |
| N status AJCC 8^th37^  N0  N1  N2 | | 31 (28.2)  45 (40.9)  34 (30.9) | 47 (30.1)  75 (48.1)  34 (21.8) | 0.234 |
| Resection margin^38^  R1 ( ≤1.0 mm) | | 45 (40.9) | 75 (48.1) | 0.262 |
| Harvested lymph nodes | | 31.0 [23.0, 39.2] | 33.0 [24.2, 42.0] | 0.533 |
| Lymph node ratio (LNR) | | 0.05 [0.00, 0.12] | 0.03 [0.00, 0.10] | 0.090 |
| Grading  Poor (G3) | | 52 (47.3) | 61 (39.1) | 0.209 |
| Perineural invasion | | 85 (77.3) | 132 (84.6) | 0.149 |
| Lymphovascular invasion | | 78 (70.9) | 112 (71.8) | 0.891 |
| Adjuvant Treatment  Chemotherapy alone  Chemoradiation  Radiation alone | | 70 (63.6)  29 (26.4)  34 (30.9)  7 (6.4) | 109 (69.9)  47 (30.1)  54 (34.6)  8 (5.1) | 0.292  0.876 |
| First recurrence type  Local-only  Liver-only  Lung-only  Multiple  Other | | 21 (19.1)  21 (19.1)  10 (9.1)  12 (10.9)  14 (12.7) | 16 (10.3)  38 (24.4)  13 (8.3)  20 (12.8)  19 (12.2) | 0.625 |
| Survival (months) [median (95% CI)]  Disease-Specific Survival  Event-Free Survival | | 43 [34.7, 51.2]  24 [19.6, 28.3] | 37 [31.2, 42.7]  22 [18.6, 25.3] | 0.154  0.558 |
|  |  |  |  |  |

**Supplementary Figures**

**Supplementary Figure 1**. Comparison of disease-specific survival (DSS) (a) and event-free survival (EFS) (b) among resectable and biological borderline resectable (BBR) patients who underwent neoadjuvant treatment.


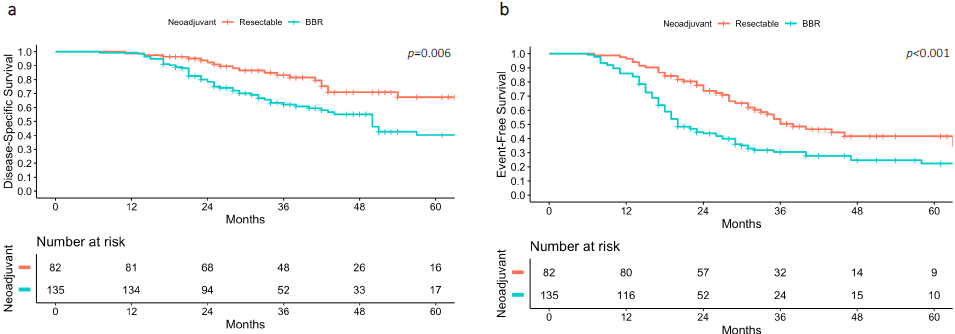


**Supplementary Figure 2**. Comparison of disease-specific survival (DSS) (a) and event-free survival (EFS) (b) among biological borderline resectable (BBR) patients receiving either upfront surgery or neoadjuvant treatment.
